# Nanoindentation experiments for single-layer rectangular graphene films: a molecular dynamics study

**DOI:** 10.1186/1556-276X-9-41

**Published:** 2014-01-22

**Authors:** Weidong Wang, Shuai Li, Jiaojiao Min, Chenglong Yi, Yongjie Zhan, Minglin Li

**Affiliations:** 1 School of Electrical and Mechanical Engineering, Xidian University, Xi'an 710071, China; 2 Physics Department, Northwest University, Xi'an 710069, China; 3 School of Mechanical Engineering and Automation, Fuzhou University, Fuzhou 350108, China

**Keywords:** Molecular dynamics simulation, Nanoindentation, Rectangular graphene film, Young’s modulus, Strength

## Abstract

A molecular dynamics study on nanoindentation experiments is carried out for some single-layer rectangular graphene films with four edges clamped. Typical load–displacement curves are obtained, and the effects of various factors including indenter radii, loading speeds, and aspect ratios of the graphene film on the simulation results are discussed. A formula describing the relationship between the load and indentation depth is obtained according to the molecular dynamics simulation results. Young’s modulus and the strength of the single-layer graphene film are measured as about 1.0 TPa and 200 GPa, respectively. It is found that the graphene film ruptured in the central point at a critical indentation depth. The deformation mechanisms and dislocation activities are discussed in detail during the loading-unloading-reloading process. It is observed from the simulation results that once the loading speed is larger than the critical loading speed, the maximum force exerted on the graphene film increases and the critical indentation depth decreases with the increase of the loading speed.

## Background

The perfect graphene is a kind of carbonaceous material which consists of two-dimensional honeycomb lattice structures of single layer of carbon atoms. It is the basic unit to build other dimensional carbonaceous materials, such as zero-dimensional fullerenes, one-dimensional carbon nanotubes, and three-dimensional graphite [1,2]. Graphene sheets/ribbons/films have attracted the interest of the scientific community because of recent exciting experimental results [3-6]. Their growth, atomic makeup, electronics, doping, and intercalation have attracted many investigations [7-10]. A suspended graphene sheet [1,11] can be used in a variety of ways, such as for pressure sensors or gas detectors [12] or mechanical resonators [13].

It is still debatable whether a graphene sheet is truly a two-dimensional structure or if it should be regarded as a three-dimensional structure since it exhibits a natural tendency to ripple, as observed in recent experiments [2,14-16]. Carlsson addressed that an understanding of the coupling behaviors between bending and stretching of graphene sheets is necessary to fully explain the intrinsic ripples in a graphene sheet [15]. In addition to theoretical investigations, recent research has been carried out to measure the mechanical properties of suspended graphene sheets by utilizing an atomic force microscope (AFM) [17]. Through weak van der Waals forces, graphene sheets were suspended over silicon dioxide cavities where an AFM tip was probed to test its mechanical properties. Their Young’s modulus differs from that of bulk graphite. Poot and van der Zan [18] measured the nanomechanical properties of graphene sheets suspended over circular holes by using an AFM and suggested that graphene sheets can sustain very large bending and stretching prior to the occurrence of fracture, which indicates that the classical Kirchhoff plate theory used in the bending and vibration analysis of graphene sheets may not be suitable since deflection and stretching are considerable [19]. Some researchers thought that the large deflection plate theory of von Kármán may be a better candidate to model the graphene sheet, and they have characterized its bending and stretching through that theory [20,21]. Lee et al. measured Young’s modulus and the maximum stress of graphene by using an AFM in the nanoindentation experiment [22] and reported the effect of grain boundaries on the measurement of chemical vapor-deposited graphene [23]. Fang et al. [24] has studied the mechanical behavior of a rectangular graphene film under various indentation depths, velocities, and temperatures using molecular dynamics (MD) simulations. The physical models of the rectangular graphene film established by Fang et al. are doubly clamped using a bridge-type support and are loaded by a flat-bottomed diamond tip.

Although the above research has been carried out for mechanical properties of graphene, the variation of the values of elastic modulus has not yet reached a consensus. Especially for rectangular graphene films, the relationship between the load and the indentation depth is not clear. Furthermore, there are few papers available which describe the deformation mechanisms and dislocation activities of graphene film during the nanoindentation processes in detail. These investigations are concentrated on tension deformation [25-28] and shear deformation [29]. Almost all of the available literatures on dislocation activities in graphene focus on theoretical studies and numerical simulations, including density functional theory (DFT) [26], tight-binding molecular dynamics (TBMD) [30], *ab initio* total energy calculation [30], and quantum mechanical computations [31]. Researchers always artificially applied defects or dislocations and then studied their effects on the properties and activities in graphene. However, due to the bottleneck of experimental study at nanoscale, a very few experimental observations of dislocation activities are available at present. Warner et al. [32] also reported the observation of dislocation pairs through HRTEM experiments and gave five possible mechanisms that describe how these dislocation pairs could have formed, namely, during the CVD growth, electron beam sputtering of carbon dimers along a zigzag lattice direction, from surface adatom incorporation, from a monovacancy, and from a Stone-Wales defect. They then concluded that edge dislocations result in substantial deformation of the atomic structure of graphene, with bond compression or elongation of ±27%, plus shear strain and lattice rotations.

In this article, some MD simulations of nanoindentation experiments are performed on a set of single-layer rectangular graphene films with four clamped edges. The dislocation activities and the deformation mechanism are discussed, and a formula is introduced in order to describe the relationship of load and indentation depth and to measure the mechanical properties of graphene.

## Methods

**Figure 1:**
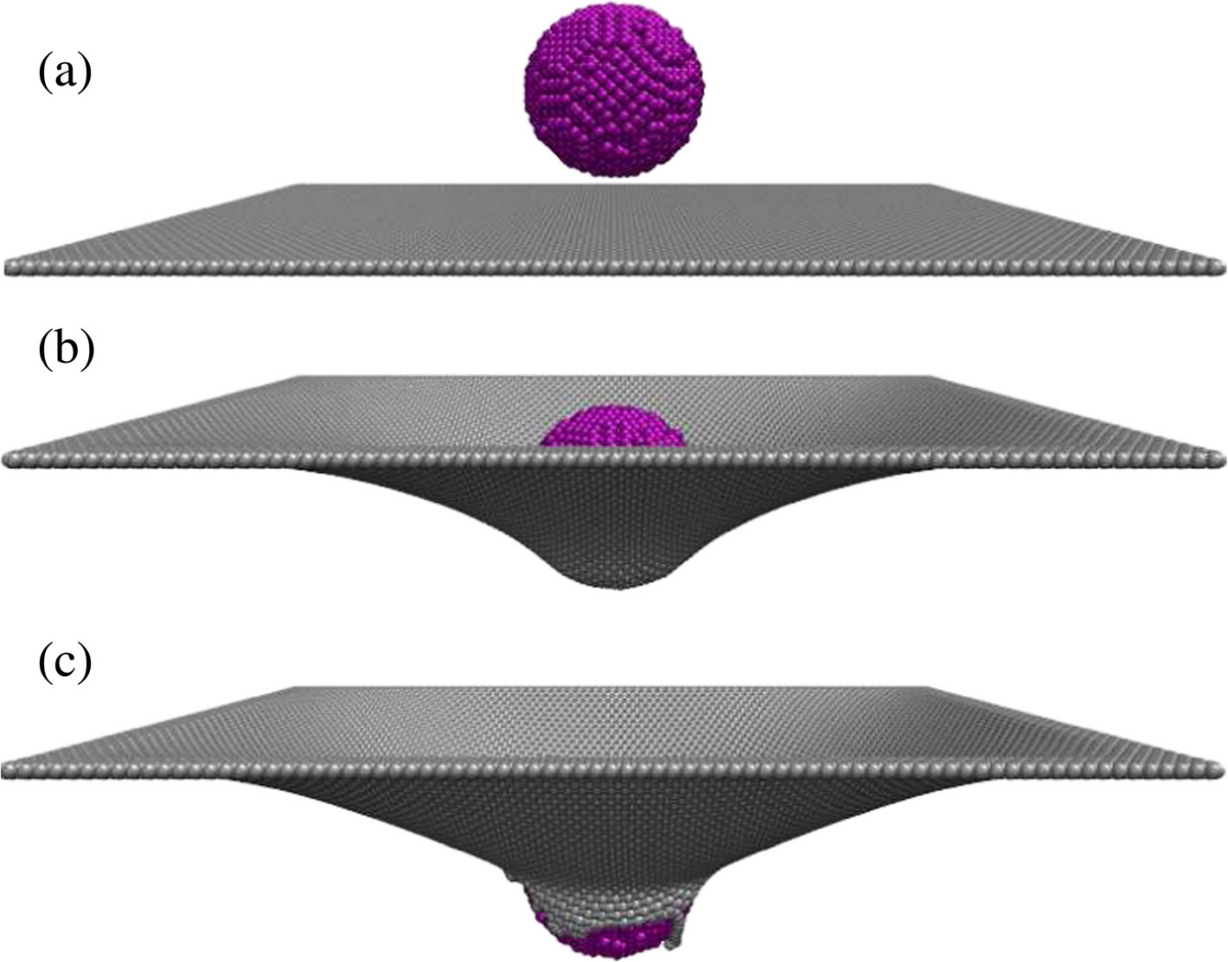
**Atomic configuration of the system model during the nanoindentation experiment. (a)** The origin model, **(b)** the state during the loading process, and **(c)** at rupture state.

In order to carry out the nanoindentation experiments, one diamond sphere was introduced to simulate the indenter. Figure 1a shows the origin model for the nanoindentation experiment. Here, the upper ball is the indenter and constructed by diamond, which is considered as a rigid object so that the atomic configuration of the diamond indenter had no changes during MD simulations. The lower plane is a single-layer rectangular graphene film with different aspect ratios. For the inner atoms of the indenter and the graphene film, the energy function was described by adaptive intermolecular reactive empirical bond order (AIREBO) potential. Compared with the Tersoff-Brenner potential, the AIREBO potential not only introduces multi-body potential effects and the local atomic circumstance effect but also adds long-range interactions and a torsion term [33], and it has been found be more suitable than the Tersoff-type potential for accurately capturing Young’s modulus of graphene as well as bond breaking and reforming between carbon atoms [28]. In order to avoid the influence of nonphysical explanations with improper cutoff functions on the fracture process, the cutoff parameter of the AIREBO potential is set to be 2.0 Å. As for the interaction between the indenter and the graphene film, van der Waals forces were simulated based on the Lennard-Jones potential.

When performing MD simulations, we use the canonical (i.e., NVT) ensemble and control the temperatures at an ideal temperature of 0.01 K. In order to avoid the complex effects of the atomic thermal fluctuations, the temperature is regulated with the Nosé-Hoover method and the time step was set to 1 fs. During the simulation, one key step, named energy minimization and relaxation, should be carried out to make the system remain in the equilibrium state with lowest energy. Then, the indentation experiment was executed and the simulation results were output for further research.

## Results and discussion

### Loading and unloading properties

We take the case of the graphene film with an aspect ratio of 1.2 and the diamond indenter with a radius of 2 nm as an example to describe the indentation experiment in the following. The indenter was placed over the geometric center of the graphene film and forced to move in the direction perpendicular to the original graphene surface. Figure 1 gives the atomic configurations of the system model during the indentation experiment at a speed of 0.20 Å/ps. The atoms on the edge of the graphene film remained in a static state due to fixed boundary conditions. After enough loading time, the graphene film is eventually pierced through by the indenter, appearing some fractured graphene lattices. The load–displacement curves can be attained from the data of intender load (*F*) and indentation depth (*d*) calculated in MD simulations. The moment the load–displacement curve drops suddenly is considered to be a critical moment. In our simulations, the load suddenly decreased once the indentation depth exceeded 5.595 nm, defined as the critical indentation depth (*d*_c_), and the corresponding maximum load (*F*_max_) is 655.08 nN.

**Figure 2:**
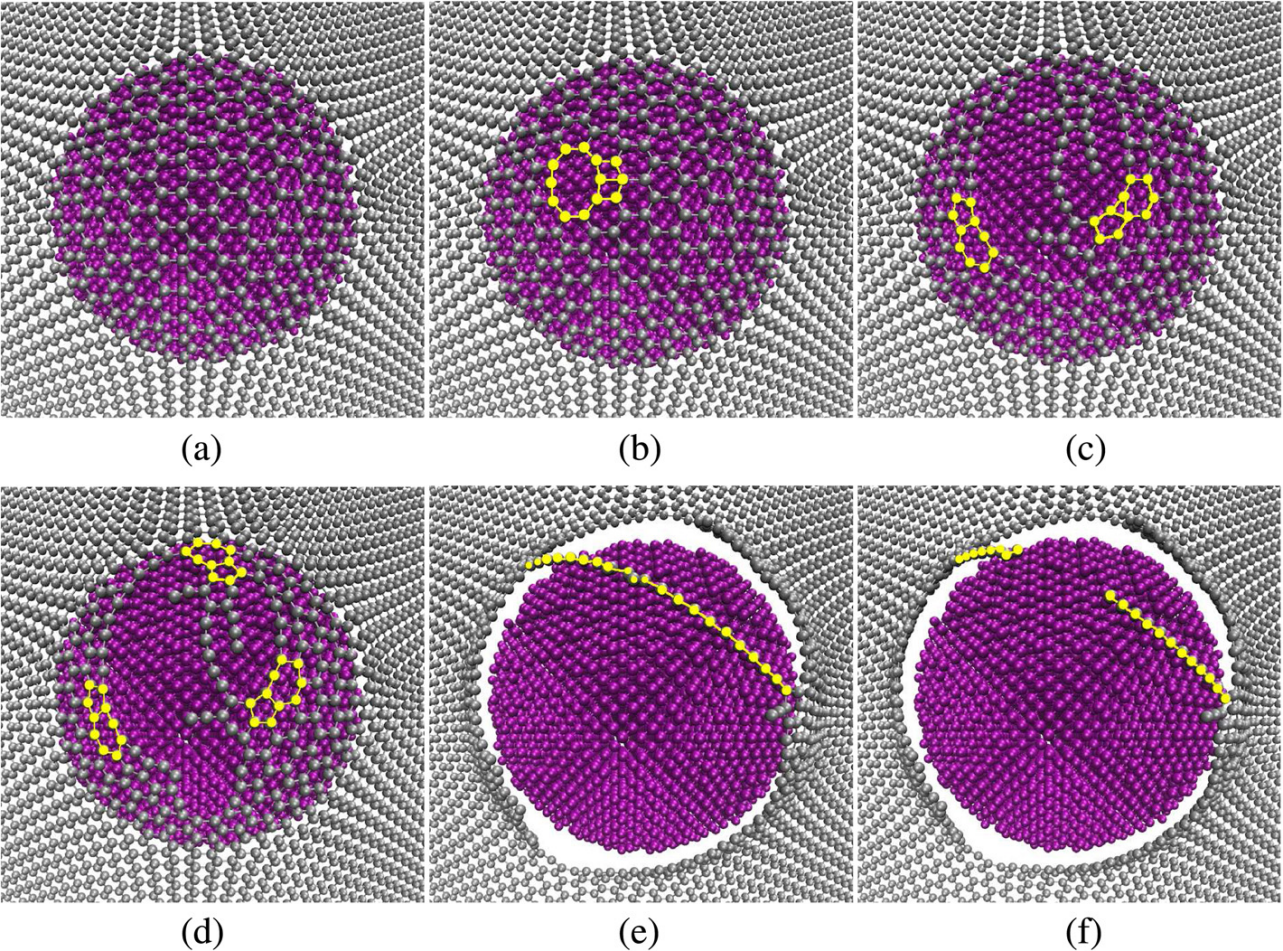
**Evolution of graphene lattice fracture at different indentation depths.** This group of figures shows the process from the state at which the indentation depth reaches the critical depth to the state the graphene film is totally ruptured with an indenter radius of 2 nm, loading speed of 0.20 Å/ps, and aspect ratio of 1.2. **(a)** At critical moment: indentation depth 55.95 Å, load 655.08 nN; **(b)** first broken bond emerged: indentation depth 55.97 Å, load 635.60 nN; **(c)** pentagonal-heptagonal (5–7) and trilateral structures emerged: indentation depth 55.99 Å, load 426.04 nN; **(d)** three 5–7 structures: indentation depth 56.01 Å, load 310.45 nN; **(e)** single-chain structure emerged: indentation depth 56.51 Å, load 112.03 nN; **(f)** fracture of the chain: indentation depth 56.61 Å, load 93.70 nN.

Figure 2 gives some detailed views on the graphene lattice fracture process starting from the critical moment. It is shown in Figure 2a that the carbon network was expanded largely, but there is no broken carbon-carbon (C-C) bond at the critical moment. Figure 2b represents the moment the bond-broken phenomenon emerged for the first time, with a pore appearing. The bond-broken process is irreversible and the load exerted on the graphene firstly declines. The first appearance of the pentagonal-heptagonal (5–7) and trilateral structures is shown in Figure 2c. Unlike with other Stone-Wales 5-7-7-5 (SW) defects mentioned in [34-36], in our indentation simulations, the loads are applied in the direction perpendicular to the surface of the graphene film and the 5–7 structure seems not conjugate but singular. In addition, it is found that the trilateral structure is an interim state in the evolution process from a pristine hexagonal structure to the 5–7 structure. A 5-3-6 structure including this trilateral structure and its adjacent structures would evolve into another 5–7 structure, the right one in Figure 2d, through bond breaking and bond reforming. Furthermore, a single-chain structure, shown in Figure 2e, can be observed during the fracture process, which can also be found in [26]. Afterwards, the single chain was broken and the indenter totally pierced through the graphene film.

**Figure 3:**
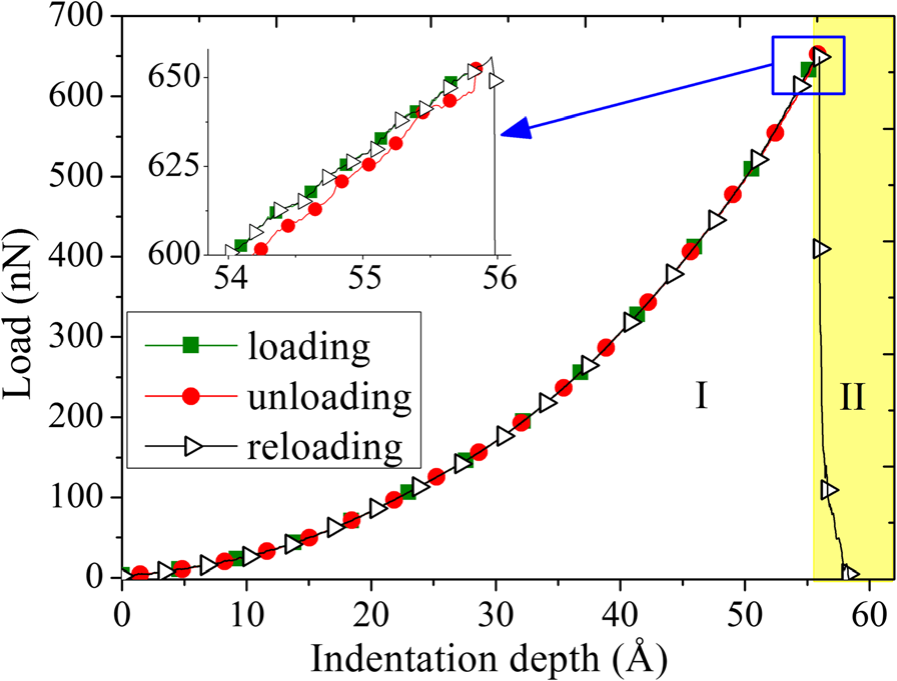
Load–displacement curve of loading-unloading-reloading process with maximum indentation depth smaller than the critical indentation depth.

**Figure 4:**
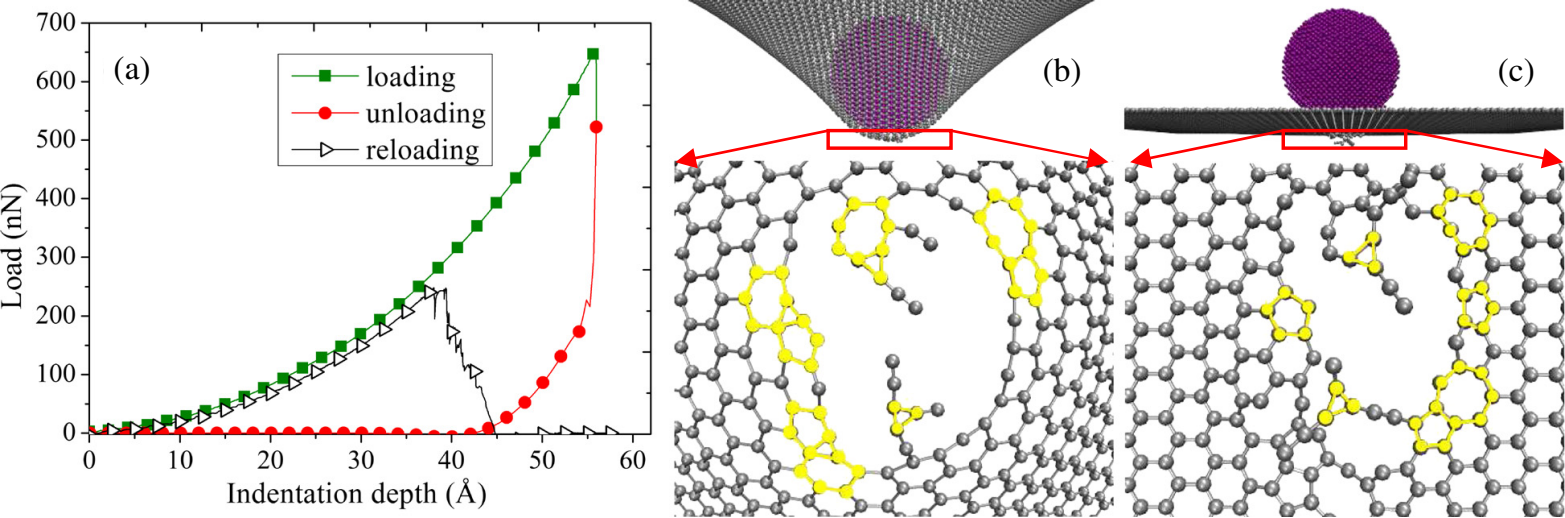
**Loading-unloading-reloading process with the maximum indentation depth smaller than the critical indentation depth. (a)** Load–displacement curve, **(b)** local atom configuration when the loading process is finished, and **(c)** local atom configuration when the unloading process is finished.

Generally speaking, elastic deformation which is reversible and plastic deformation which is irreversible are two typical kinds of deformation of an object or material in the view of engineering. In order to determine whether the deformation of the graphene film is elastic or plastic, a set of experiments of loading-unloading-reloading processes are conducted. As shown in Figure 3, during the continuous loading process of the indenter on the graphene film, it can be found that the graphene film mainly takes on two stages in sequence:

• Stage I. The unloading process is done before the indentation depth reaches the critical depth, *d*_c_. The graphene sheet almost can make a complete recovery, i.e., restore its initial structures, and the curves of reloading processes almost perfectly match the initial loading curve while the unloading curve shows very small deviations from the initial one, as shown in the inset of Figure 3. In general, the almost-perfect coincidence is due to the fact that the carbon covalent bonds and the graphene lattice structure are not destroyed. It can be concluded that there is no plastic deformation in this stage, i.e., the graphene undergoes elastic deformation.

• Stage II, i.e., the yellow region in Figure 3. In Figure 4a,b,c, it is after the indentation depth exceeded *d*_c_ that the unloading process begins. The graphene sheet cannot make a complete recovery, and there exited broken covalent bonds after the unloading process. In the reloading process, the maximum force exerting on graphene is much smaller than that in Figure 3, which denotes the fracture of graphene lattices. Figure 4b describes the state where the unloading process begins, and Figure 4c describes the state where the unloading process ends. After the loading process, there exited broken bonds and fractured lattices in the middle of the graphene film and these defective structures did not recover during the unloading process. Therefore, the deformation of the graphene described in this figure can be considered as a plastic type.

### Young’s modulus and strength of the graphene film

According to the available correlation for the indentation experiments of a circular single-layer graphene film in [18,22,37], one new formula is constructed to describe the relationship between indention depth and load,

(1) $$F=F_{\sigma }\left(d\right)+F_{E}\left(d\right)$$

where *d* is the indentation depth and *F* denotes the concentrated force gotten by the graphene film. In Equation 1, the load *F* consists of two parts: the first part, *F*_*σ*_(*d*), represents the term due to the axial tension of the two-dimensional (2-D) film,

(2) $$F_{\sigma }\left(d\right)=\sigma_{0}^{2D}\left(\mathrm{\pi R}\right)\beta^{1/2}{\left(\frac{r}{R_{\mathrm{equ}}}\right)}^{3/4}\left(\frac{d}{R_{\mathrm{equ}}}\right)$$

where *σ*_0_^2D^ is the pre-tension of the single-layer graphene film, *r* is the indenter radius, *β* denotes the aspect ratio and is equal to *L*/*b*, and *R*_equ_ represents the equivalent radius of the rectangular graphene sheet, (*Lb*/*π*)^1/2^. The second one, *F*_E_(*d*), represents the large deformation term,

(3) $$F_{E}\left(d\right)=E^{2D}\left(q^{3}R_{\mathrm{equ}}\right)\beta^{1/2}{\left(\frac{r}{R_{\mathrm{equ}}}\right)}^{1/4}{\left(\frac{d}{R_{\mathrm{equ}}}\right)}^{3}$$

where *E*^2D^ is the 2-D elastic modulus, i.e., Young’s modulus, of the single layer graphene film. The strain energy density of graphene, as a standard 2-D material, can be represented by the energy of per unit area. Then, the corresponding pre-tension and elastic modulus can be expressed as *σ*_0_^2D^ and *E*^2D^, respectively, with the unit N/m. The common pre-tension and elastic modulus of a 3-D bulk material can be obtained through these 2-D values divided respectively by the effective thickness which is always treated as the layer spacing of the graphite crystal, i.e., 3.35 Å. *q* is an nondimensional value, *q* = 1/(1.05 - 0.15*ν* - 0.16*ν*^2^) = 0.9795, where *ν* denotes Poisson’s ratio, *ν* = 0.165 [3,18,21]. It is reported that when *r*/*R* > 0.1, the indenter radius has a significant influence on the load–displacement properties [38,39]. In our simulations, *r*/*R* > 0.1; thus, Equations 2 and 3 are corrected by a factor of (*r*/*R*)^3/4^ and (*r*/*R*)^1/4^, respectively.

According to the expression of maximum stress in the plane of a circular elastic film in [40], the maximum stress for a rectangular elastic film can be expressed by the following formula:

(4) $$\sigma_{m}^{2D}={\left(\frac{F_{\max}E^{2D}}{8\mathrm{\pi r}}\right)}^{1/2}$$

**Figure 5:**
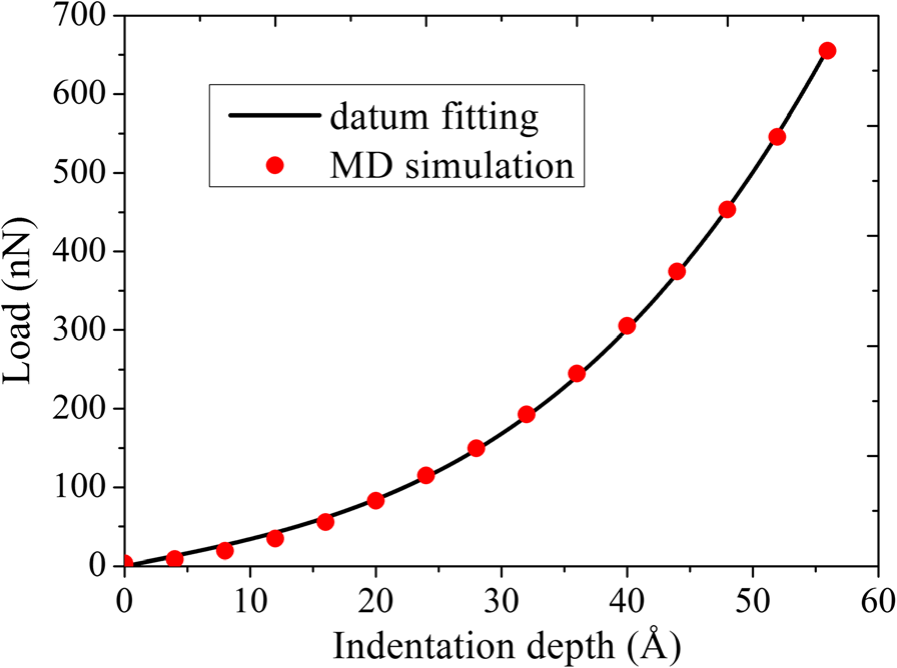
Curves of indentation depth versus load for the nanoindentation experiment.

Figure 5 presents the curves of indentation depth versus load for the nanoindentation experiment. The red solid curve is from the MD simulation results. According to Equation 1, nonlinear least squares method was used to fit the simulation results, and then the black curve in Figure 5 can be obtained. It is noted that when the indentation depth is about 5.597 nm, the load received by the graphene film suddenly drops from approximately 655.08 to approximately 522.172 nN. Corresponding to Figure 2b,c, the lengths of C-C bonds under the indenter quickly become larger than before, which indicates that the bonds were broken.

Table 1 gives the mechanical properties calculated from the MD simulation results. Young’s modulus and the maximum stress of the graphene are obtained as 1.0539 TPa and 205.1328 GPa, respectively. Young’s modulus obtained in this paper is in good agreement with those obtained by both experimental and numerical methods. Kudin et al. has predicted a Young’s modulus of 1.02 TPa using *ab initio* methods [41]. Lee et al. obtained a Young’s modulus of 1 ± 0.1 TPa by nanoindentation in an AFM of freestanding monolayer circular graphene membranes [22]. Neek-Amal and Peeters studied the nanoindentation of a bilayer graphene using molecular dynamics simulations and estimated a Young’s modulus of 0.8 TPa [42]. In addition, the maximum stress ranges from 130 to 240 GPa by means of both experiments and numerical simulations reported in other literatures [21,22,43,44]. The maximum stress obtained in this paper can also be included in the above range, which verified our simulation results. The changing trend of 2-D pre-tension demonstrates that the pre-tension of the rectangular graphene film is positively correlated with the loading speed of the indenter. The indenter size also affects the pre-tension, which, to some extent, explains why the correction factors were introduced in Equations 2 and 3.

**Table 1:** Mechanical properties of the single-layer graphene film from nanoindentation experiments

**Indenter radius (Å)/speed (Å/ps)**	**2-D elastic modulus (N/m)**	**3-D elastic modulus (TPa)**	**2-D pre-tension (N/m)**	**3-D pre-tension (GPa)**	**2-D max stress (N/m)**	**3-D max stress (GPa)**
10/0.10	375.0644	1.1196	38.8546	115.9840	72.4895	216.3866
10/0.20	375.0096	1.1194	38.8589	115.9966	72.4771	216.3496
20/0.10	335.0012	1.0000	28.5092	85.1021	66.1326	197.4106
20/0.20	335.2572	1.0008	28.4879	85.0385	66.0994	197.3115
30/0.10	349.1828	1.0423	22.7998	68.0590	67.4504	201.3445
30/0.20	348.8383	1.0413	23.0197	68.7154	67.6680	201.9940
Average	353.0589	1.0539	/	/	68.7195	205.1328

### Other parameters’ influences on nanoindentation experiments

**Figure 6:**
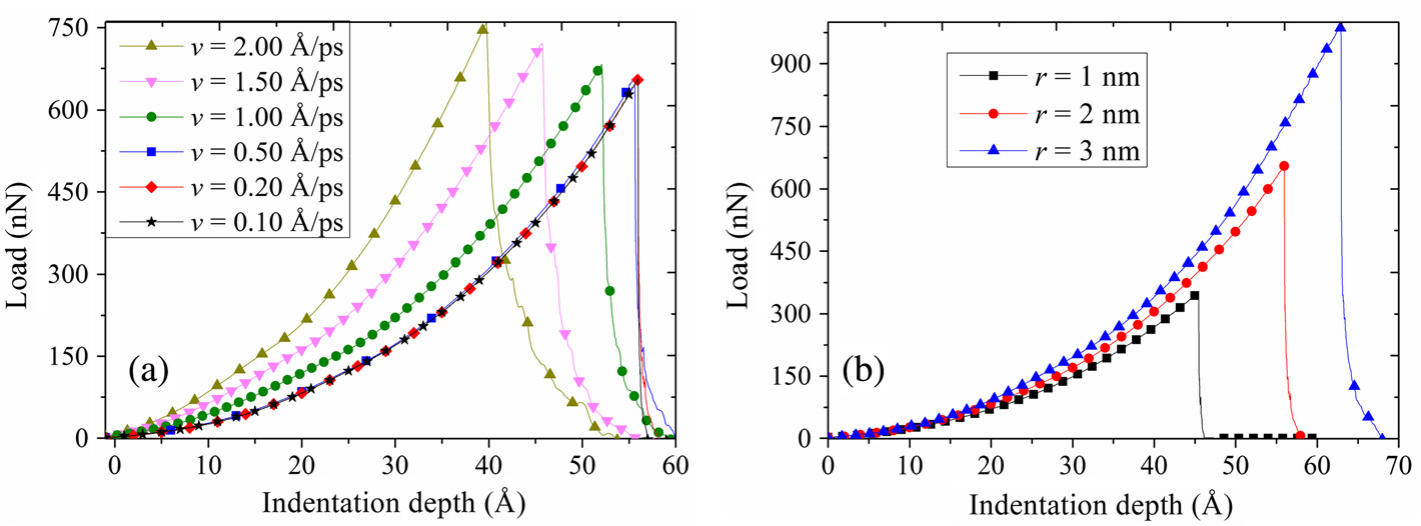
**Comparison of load versus indentation depth for different parameters. (a)** The indenter is loaded at different loading speeds between 0.10 and 2 Å/ps. **(b)** The indenter is loaded with different indenter radii of 1, 2, and 3 nm.

**Figure 7:**
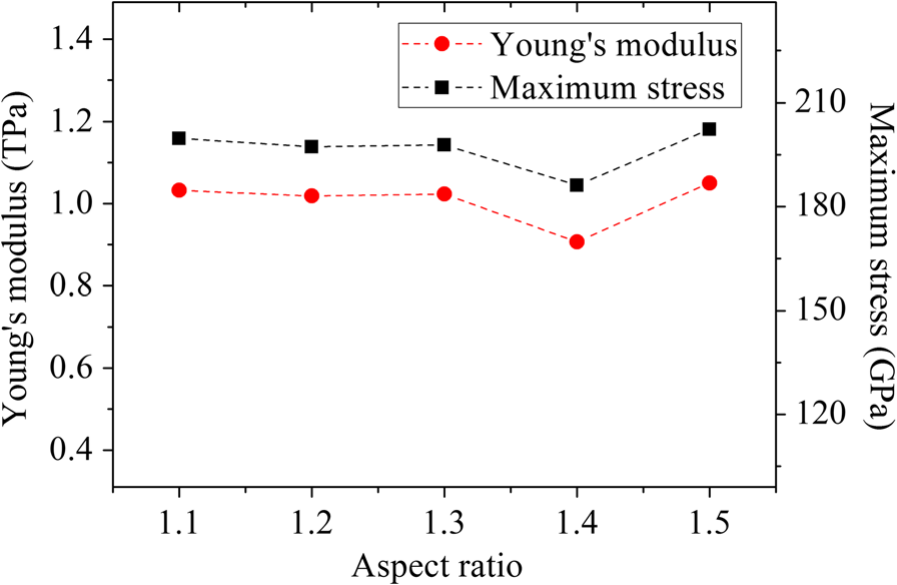
Comparison of Young’s modulus and maximum stress versus the aspect ratio of the graphene film.

For further study of nanoindentation properties, a series of simulations have been carried out with different loading speeds, indenter radii, and aspect ratios of graphene film. It is indicated that the speed of 0.20 Å/ps can be regarded as the critical loading speed, as shown in Figure 6a. When the loading speed is higher than the critical value, with the increase of speed, the maximum load increases rapidly; simultaneously, the critical indentation depth decreases rapidly. However, when the loading speed is lower than the critical value, the changes of *F*_max_ and *d*_*c*_ are not that obvious. When the loading speed decreases from 1.00 to 0.50 Å/ps, dropping by 50%, the value of *d*_c_ increases by 33.35%, and the value of *F*_max_ decreases by 8.43% correspondingly. Nevertheless, when the loading speed decreases from 0.20 to 0.10 Å/ps, dropping by 50%, the changes of *F*_max_ and *d*_c_ are only 1.68% and 0.21%, respectively. The results may be attributed to the fact that the higher the loading speed of the indenter, the less time it takes to go through the graphene sheet, resulting in a higher load and lower indentation depth than those at a lower loading speed, in which situation the load process is much slower. Secondarily, the spherical indenter’s influences on results are observed by changing the indenter radius. The simulations of various indenter radii (1, 2, and 3 nm) are carried out at the speed of 0.20 Å/ps. The results of the load–displacement curve are shown in Figure 6b. The stress is more uniform in the middle of the graphene, so the *F*_max_ increases obviously and the critical indentation depth also becomes greater with the increase of the indenter radius. Finally, after changing the aspect ratio of the graphene film to 1.1 and 1.5, Young’s modulus and the maximum stress of the graphene are obtained using the methods mentioned above. It can be deduced from Figure 7 that Young’s modulus and the maximum stress are the inherent properties of graphene and irrelevant to its size, which also verifies the formula obtained above.

## Conclusions

Some MD simulations of nanoindentation experiments on single-layer rectangular graphene sheets have been carried out in order to obtain the mechanical properties of graphene. A correlation between the load and the indentation depth is constructed, and Young’s modulus and the strength of graphene are obtained in the end. The simulation results show that the unloaded graphene film could make a complete recovery if the maximum indentation depth is less than the critical indentation depth, and the graphene film undergoes elastic deformation during the whole loading-unloading-reloading process. However, if the maximum indentation depth is larger than the critical indentation depth, the graphene sheet could not restore its original structures after unloading and the graphene deforms plastically. Based on the simulations of nanoindentation at different loading speeds and indenter radii, it can be observed that the maximum load increased and the critical indentation depth decreased with the increase of loading speed. In addition, the indenter radius has a remarkable influence on the force-displacement curve. As the indenter radius increases, the critical load and the critical indentation depth also increase.

## Abbreviations

2-D: two-dimensional; AFM: atomic force microscope; AIREBO: adaptive intermolecular reactive empirical bond order; GPa: gigapascals; MD: molecular dynamics; NVT: constant number of particles, volume, and temperature; SW: Stone-Wales 5-7-7-5; TPa: terapascals.

## Competing interests

The authors declare that they have no competing interests.

## Authors’ contributions

The analysis of the simulation results was mainly carried out by WDW. The simulation processes were mainly conducted by SL, JJM, and CLY. Some fairly helpful proposals about the construction of models were made by YJZ and MLL. All authors read and approved the final manuscript.
