# Correction: Nanoindentation experiments for single-layer rectangular graphene films: a molecular dynamics study

**DOI:** 10.1186/1556-276X-9-228

**Published:** 2014-05-10

**Authors:** Weidong Wang, Shuai Li, Jiaojiao Min, Chenglong Yi, Yongjie Zhan, Minglin Li

**Affiliations:** 1 School of Electrical and Mechanical Engineering, Xidian University, Xi’an 710071, China; 2 Physics Department, Northwest University, Xi’an 710069, China; 3 School of Mechanical Engineering and Automation, Fuzhou University, Fuzhou 350108, China

## Correction

In the original version of this article [1], the sizes of the simulated graphene models with different aspect ratios were not listed. Here, we add Table one (Table 1 here) to illustrate the sizes of rectangular graphene films simulated in the article [1].

**Table 1:** Sizes of rectangular graphene films with different aspect ratios

**Length**	**Width**	**Aspect ratio**
181	121	1.5
181	129	1.4
181	139	1.3
181	149	1.2
181	159	1.1

In addition, there is a typo in the Acknowledgements section of the origin version of this article [1]. The right one is given in the following.

## Acknowledgements

We acknowledge the financial support provided by the Natural Science Basic Research Plan in Shaanxi Province of China (grant no. 2013JM7017), subsequently by the National Natural Science Foundations of China (grant no. 51205302 and no. 50903017) and the Fundamental Research Funds for the Central Universities in Xidian University (grant no. K5051304006). We also would like to thank all the reviewers for their comments and kind suggestions to our manuscript and all the editors for their careful corrections on the final version of the article.
